# Moderately Increased Left Ventricular Filling Pressure Suggesting Early Stage of Heart Failure with Preserved Ejection Fraction in Patients with Invasively Assessed Coronary Microvascular Dysfunction

**DOI:** 10.3390/jcm13226841

**Published:** 2024-11-14

**Authors:** Jacek Arkowski, Marta Obremska, Przemysław Sareło, Magdalena Wawrzyńska

**Affiliations:** 1 Pre-Clinical Research Center, Wrocław Medical University, Karola Marcinkowskiego 1, 53-368 Wroclaw, Polandmagdalena.wawrzynska@umw.edu.pl (M.W.); 2 Klodzko County Hospital, Szpitalna 1, 57-300 Klodzko, Poland; 3 Institute of Heart Diseases, Wrocław Medical University, Borowska 213, 50-556 Wrocław, Poland; marta.obremska@umw.edu.pl; 4 Department of Biomedical Engineering, Faculty of Fundamental Problems of Technology, Wrocław University of Science and Technology, Wybrzeże Wyspiańskiego 27, 50-370 Wrocław, Poland

**Keywords:** coronary microvascular disfunction, HFpEF, IMR, CFR, strain

## Abstract

**Background**: With modern diagnostic tools, incidence ischemia with no obstructive coronary atherosclerosis (INOCA) and heart failure with preserved ejection fraction (HFpEF) are found to be much higher than previously believed, and—as they lead to adverse cardiovascular outcomes—their causes and development are subjects of ongoing research. There is growing evidence that coronary microvascular dysfunction might be the underlying cause of both INOCA and HFpEF. **Methods**: In 65 patients with effort angina but no obstructive coronary artery disease, the index of microvascular resistance and coronary flow reserve were measured invasively in the LAD. The echocardiographic parameters, including left atrial strain, left ventricular strain, and indices of left ventricular diastolic dysfunction, were compared between two groups of patients: those with normal coronary microcirculation parameters and those with impaired coronary microvascular function. **Results**: Patients with coronary microvascular dysfunction had higher a E/E′ index than those with normal microvessel reactivity. This finding was further confirmed by ROC analysis. The groups did not differ significantly in values of other echocardiographic parameters, including the left ventricular and left atrial strain. The prevalence of classical cardiovascular risk factors was similar in both groups. **Conclusions**: The coexistence of impaired coronary microvascular function with moderately elevated left ventricular filling pressures might correspond to the co-development of early stages of coronary microvascular dysfunction and HFpEF.

## 1. Introduction

In many patients with exertional angina, there is no evidence of obstructive coronary artery disease in angiography. This clinical presentation, known as INOCA, poses many challenges for treating physicians. It is often hard to determine the primary underlaying cause of the symptoms and to choose an effective treatment. INOCA is not a benign disease, as it is associated with recurrent chest pain, impaired functional capacity, reduced health-related quality of life, and high healthcare costs. It is also increasingly being recognized as a substantial contributor to adverse cardiovascular mortality and outcomes, including myocardial infarction and heart failure with preserved ejection fraction (HFpEF).

### 1.1. Coronary Microvascular Dysfunction

The assessment of vessel function helps to explain the symptoms and, at least theoretically, offers some rationale for treatment. Abnormal epicardial coronary vascular function and coronary microvascular dysfunction (CMD) have been identified in the majority of INOCA patients on invasive coronary function testing. Impaired vessel reactivity can be divided into two broad categories: inability to dilate and thus increase coronary flow and a tendency to vasoconstriction. The first phenomenon is best determined by invasive measurement of coronary flow reserve (CFR) and index of microvascular resistance (IMR). Higher hyperemic microvascular resistance is associated with lower CFR (i.e., presence of CMD). CMD through endothelium-dependent and independent mechanisms contributes to adverse outcomes in INOCA [1] and identifies patients with higher-risk INOCA [2].

CMD is currently thought to be a consequence of submicroscopic changes in the structure of both myocardium and microvessels, leading to altered function of heart muscle and arterioles (narrowing, perivascular fibrosis, and capillary rarefication). While all predisposing factors are not clearly understood, it has been suggested that cardiometabolic risk factors, increased oxidative stress, and inflammation are the most probable causes of CMD and INOCA [3].

CMD may be assessed non-invasively using transthoracic Doppler echocardiography, positron emission tomography, cardiac magnetic resonance, or cardiac computer tomography. Each of these modalities has its drawbacks (cost, radiation exposure, indirect measurements, or a combination of those factors) [4].

Invasive testing of coronary microvascular function is currently believed to be the gold standard. The measurements can be obtained by assessing flow velocity by Doppler or using the thermodilution method.

### 1.2. HFpEF and Diastolic Dysfunction

In recent times, much attention has been brought to echocardiographic findings that may reveal early stages of ischemic heart disease in patients with angina symptoms and no obstructive coronary disease. In patients with preserved left ventricular systolic function, several echocardiographic parameters have been proposed as markers of early changes in myocardial function. The most important of them include diastolic dysfunction of the left ventricle and abnormal strain of both the left ventricle and left atrium.

Heart failure with preserved ejection fraction (HFpEF) is a common clinical syndrome that is increasing in prevalence. LV diastolic dysfunction plays a crucial role in the development of HFpEF [5]. LV diastolic dysfunction is defined by impaired relaxation, an increase in chamber stiffness, or some combination of the two. Elevated filling pressures result in dyspnea, impaired exercise capacity, an increased risk for heart failure hospitalization, and decreased survival in HFpEF [6]. In some patients with diastolic dysfunction, HFpEF does not develop. Several other abnormalities, including subtle LV systolic dysfunction, LA impairment, and CMD, are postulated to play an additional role in symptomatic HFPEF [7]. Diastolic dysfunction is usually defined as the combination of several echocardiographic parameters. The elevated E/E′ index is one of the most important of them as it reflects LV filling pressure. It has been shown to predict prognosis in patients with HFpEF [7,8,9].

### 1.3. Left Ventricular and Left Atrial Strain

Global longitudinal strain (GLS) is a crucial parameter in assessing LV function [10,11,12]. It is measured in order to determine the risk, prognosis, and treatment response of patients with heart failure [13]. It has been suggested that an impaired GLS in patients with chest pain syndrome, non-obstructive CAD, and CMD reflects subclinical LV systolic dysfunction and lack of LV contractile reserve due to underlying myocardial ischemia [14]. Left atrial function may help to determine risk and prognosis in various cardiovascular diseases [15,16]. Left atrial strain may detect subclinical myocardial dysfunction [17].

In the presented study, we aimed to investigate whether abnormal coronary microvascular function evaluated by invasive measurements of IMR and CFR is associated with any echocardiographic abnormalities that are frequently found in HFpEF, especially in its early stages. The parameters of interest were global strain of the left ventricle and left atrium measured by speckle tracking echocardiography as well as parameters of LV diastolic function assessed by Doppler. We expected the findings to offer some insight into the pathophysiology of both microvascular angina and HFpEF, especially in the early stages of possible co-development of these conditions.

## 2. Materials and Methods

The study included 65 consecutive patients referred to an invasive angiography due to effort angina, in whom no obstructive CAD in the LAD (defined as no angiographic stenosis > 50% and/or FFR < 0.80) was found.

Patients with either of the following:

- History of anterior MI;
- Heart failure with reduced ejection fraction;
- Moderate or severe valvular heart disease;
- Severe co-morbidities (e.g., malignancies, advanced respiratory, renal, or liver failure).

Were excluded from the study.

Heart failure with mildly reduced ejection fraction was not an exclusion criterion.

### 2.1. Invasive Assessment of Coronary Flow Reserve and Index of Microvascular Resistance

As it is routinely performed for cardiac catheterization, patients were required to fast for 6 h before the procedure. Additionally, any medication that might influence coronary vasomotion measurements (such as nitrates, beta blockers, and calcium channel blockers) was withheld in the morning before the procedure. A 6-French guiding catheter was advanced into the left coronary artery by either the femoral or the radial access, at the discretion of the operator. After intracoronary administration of 200 microgram of nitroglycerin and proper equalization of pressures, FFR was measured in the left anterior descending artery (LAD) using a pressure/temperature wire (Pressure wire X™ Abbott, Saint Paul, MN, USA). Then, a state of maximal hyperemia was induced by adenosine infusion (140 μg/kg/min—standard dosage for studies of coronary physiology). Room temperature saline was injected into the coronary artery three times consecutively to obtain reproducible and consistent thermodilution curves from which the mean transit time was calculated. The distal coronary pressure was measured by a pressure–temperature sensor. The values of IMR and CFR were calculated by Coroventis software (Abbot).

### 2.2. Echocardiography Measurements

All 65 patients underwent a comprehensive transthoracic echocardiographic examination using commercially available equipment (Siemens Accuson Redwood 2.0). The echocardiographer was blinded to the invasively obtained data.

Standard 2D echocardiographic measurements were performed following the ASE/EACVI guidelines. Left ventricular ejection fraction (LVEF) was calculated using the modified biplane Simpson’s rule. Maximum LA volume indexed to body surface area (LAVI) was calculated using the biplane method of disks at end-systole.

The LV inflow parameters, including peak early diastolic flow velocity (E) and late diastolic flow velocity (A), were assessed from the apical 4-chamber view by a pulsed-wave Doppler with the sample volume placed between the tips of the mitral leaflets. The pulsed-wave tissue Doppler was used to evaluate peak early diastolic tissue velocity (e) at the septal and lateral sides of the mitral annulus. The ratio of early diastolic mitral inflow velocity (E) to the average e’ velocity from both parts of the mitral annulus (E/e′) was calculated.

Analysis of longitudinal strain was performed using speckle tracking echocardiography with semiautomatic software VA20 provided by the echocardiograph. This was performed during end-expiratory breath-hold and stable ECG recording with an adequate grayscale image, allowing proper visualization of the endocardial border. The frame rate for images was adjusted between 60 and 80 frames/s. For the analysis, three consecutive cardiac cycles were recorded and averaged. The endocardial and epicardial borders were initially traced automatically, followed by manual adjustment to ensure optimal contour tracing.

Left ventricular global longitudinal strain was presented as the average of peak longitudinal value obtained from apical three standard views (4-chamber, 3-chamber, and 2-chamber). The onset of QRS was accepted as the zero-reference point. Left atrial longitudinal strain was presented as the average value evaluated from 4- and 2-chamber views. Peak atrial longitudinal strain (PALS) was measured as the peak value of longitudinal strain during left ventricular systole, and peak atrial contractile strain (PACS) as the value of strain at the onset of the P wave on electrocardiography. All strain parameters were reported as absolute values.

The LA stiffness index was calculated using the following formula: LA stiffness = (E/e′) /PALS. All measurements were performed by a cardiologist experienced in echocardiography.

### 2.3. Statistical Methods

The normal distribution of quantitative variables was verified by the Shapiro–Wilk test. Homogeneity of category variable distribution was verified with a chi-squared test. Correlation between two quantitative variables was examined by calculating the Spearman rank correlation coefficient since some of the variables did not display normal distribution. Subsequently, it was verified whether its value is significantly different from 0, thus checking whether the two variables are correlated. In cases where for an analyzed pair of variables some observations were missing, the analysis was performed on a smaller group with complete observation of the variables analyzed. The ROC curve was also analyzed to explore the potential predictive value of the measured echocardiographic parameters for abnormal IMR or CFR.

For all the tests, the level of significance was prespecified at 0.05.

Several extreme data outliers were not included in the calculations; observations with values lower than 1.5 IQR (interquartile range) from the lower quartile or values higher than 1.5 IQR from the upper quartile were defined as outliers. Those outliers were treated as missing data in the subsequent analyses. As a consequence of the above criteria and the use of nonparametric tests, it was not necessary to perform sensitivity tests for the presence or non-presence of outliers.

The study was conducted in accordance with the Declaration of Helsinki, and the protocol was approved by the Ethics Committee of Wroclaw Medical University (Project identification code 224/23N) on 26 October 2023.

Informed consent for participation was obtained from all subjects involved in the study.

## 3. Results

### 3.1. Characteristics of the Study Group

The study group included 15 women and 50 men. It was found that 80% of patients had been previously diagnosed with arterial hypertension and 25% with diabetes. Moreover, 32% were active or former smokers. Furthermore, 73% of patients had CCS II angina and 27% were in the CCS III class. Most of them were on standard antianginal drugs (beta-blockers, 72%; calcium channel blockers, 23%; nitrates, 37%), and most of them were also on statins (81%).

The distribution of qualitative variables is presented in Table 1. The values of echocardiographic parameters and quantitative cardiovascular risk factors for groups with normal and elevated IMR are presented in Table 2. The values of echocardiographic parameters and quantitative cardiovascular risk factors for groups with normal and decreased CFR are presented in Table 3.

### 3.2. Cardiovascular Risk Factors

For most classic cardiovascular risk factors (hypertension, diabetes, obesity, and smoking history), there was no significant difference between the elevated IMR group and the normal IMR group.

Likewise, the low CFR group did not differ from the normal CFR group in terms of cardiovascular risk factors, with the notable exception of higher LDL levels in the low CFR group (110.56 and 72.70, respectively, *p* = 0.019). In addition, there was a marked trend to higher LDL levels in the elevated IMR group, but the difference did not reach statistical significance (*p* = 0.06).

### 3.3. Echocardiographic Parameters

The E/E′ was higher in patients with abnormal (low) CFR than in patients with normal CFR (8.84 and 6.69, respectively, *p* = 0.016).

There was a trend towards higher E/E′ in patients with elevated IMR (compared to those with normal IMR), although the difference did not reach statistical significance (7.43 vs. 6.91, *p* = 0.36).

The potential coexistence of elevated E/E′ with elevated IMR was further confirmed by ROC analysis.

**Figure 1:**
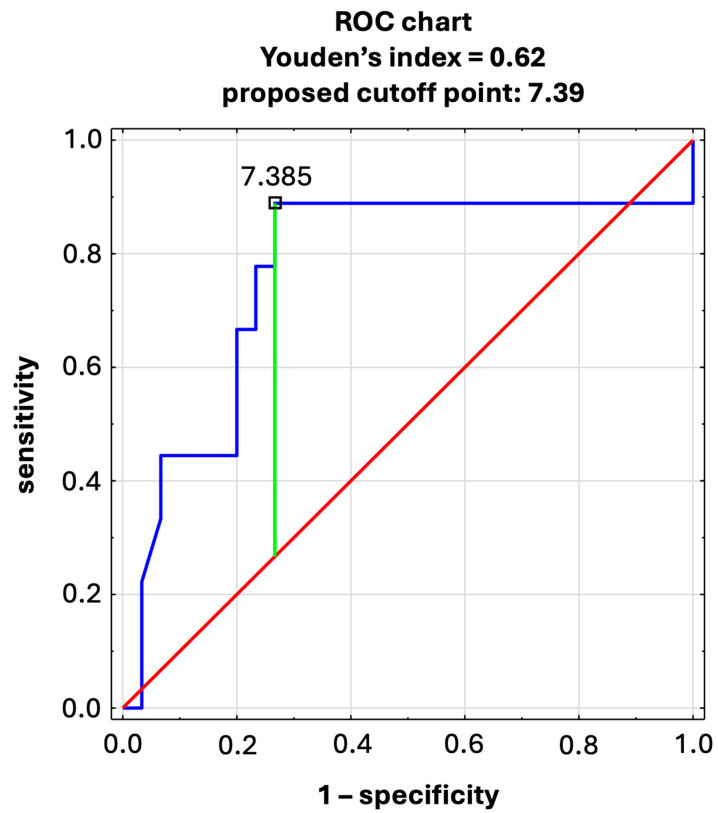
Youden’s index; E/E′ as the explanatory variable for elevated IMR (Blue—ROC curve; Red—reference line; Green—proposed cutoff point).

The ROC curve analysis suggests a statistically significant predictive value of IMR for the level of E/E′. The area under curve (AUC) suggests fairly good modeling of E/E′ by IMR level (Figure 1, Table 4).

None of the other analyzed echocardiographic parameters showed a statistically significant predictive value for either IMR or CFR.

There have been no statistically significant differences in any other echocardiographic parameters between patients with normal IMR and elevated IMR. A trend towards a higher LA stiffness index in patients with abnormal CFR was observed.

No significant correlation between coronary microcirculation function parameters (CFR and IMR) and echocardiographic strain measurements (GLS, PALS, PACS) was observed.

## 4. Discussion

### 4.1. Diastolic Dysfunction

We found significantly higher values of E/E′ (one of the most important markers of LV diastolic dysfunction) in patients with reduced CFR as well as a trend towards higher E/E′ in patients with elevated IMR (further confirmed by ROC analysis), although the values of E/E′ were not high enough to indicate increased LV filling pressure. A Youden’s index of 0.62 might suggest that moderately elevated E/E′ is a fairly good predictor of elevated IMR. From the cutoff point resulting from ROC analysis, it can be concluded that in patients with E/E′ > 7.39, an elevated microcirculatory resistance may be suspected and, perhaps, invasive testing for CMD might be justified. High E/E′ and CMD are reported by Taquetii et al. [18], who also showed that the patients with HFpEF and CMD are characterized by worse prognosis (than healthy ones or those with either HFpEF or CMD alone). Such findings strongly suggest that neither CMD nor HFpEF is a benign disease. Moreover, we observed a trend toward increased LA stiffness index in patients with reduced CFR. LA stiffness has been shown to be independently associated with HFpEF and exercise intolerance [19], and therefore our finding might be interpreted as suggesting an association between HFpEF with symptoms on exertion and an increased microvascular resistance.

The high prevalence of CMD in patients with HFpEF has been shown in several studies. Endothelium-independent abnormal coronary vasoreactivity has been reported in 66% of investigated patients with HFpEF, with even higher rates (81%) when only patients without obstructive epicardial CAD were included [20]. Similarly, 75% of HFpEF patients in Shah’s cohort were diagnosed with CMD [9]. Kopeva et al. also found a correlation between CMD (measured in SPECT) and left ventricular diastolic dysfunction [21].

As for studies with invasive measurement of microcirculation, Dryer et al. reported a higher incidence of elevated IMR in HFpEF patients than in healthy controls [22]. An association between diastolic dysfunction and invasively measured IMR was also observed by Keulards et al. [23].

### 4.2. GLS

In our cohort, the patients with normal and elevated IMR did not differ in terms of GLS values. Similarly, Michelsen et al. found no association between CMD and low GLS (although GLS reserve was reduced in CMD patients) [24].

Several studies reported reduced GLS in patients with CMD. Some of them did not measure microvascular dysfunction, defining it instead as angina with no obstructive coronary artery stenosis [25] or HFpEF with increased TIMI frame count in angiography [26]. With no quantitative assessment of either CFR or IMR, it may be hypothesized that those groups included patients with angina due to causes other than CMD or obstructive CAD. Consequently, those results cannot be directly compared to ours, where invasive testing for CMD was carried out. TIMI frame count may be interpreted as microvascular dysfunction, but this indirect assessment is potentially less reliable than our invasive measurement.

Association of CMD with abnormal LV strain has also been observed in studies with Doppler TTE used to asses CMD. Such results were seen in the Promis HF study (lower LV, lower LA strain, and more adverse outcomes in CMD patients) [27] as well as in a cohort of HfpEF patients investigated by Shah et al. (LV longitudinal systolic, LA, and right ventricular strain, all lower in the CMD group and interpreted as subendocardial abnormalities) [9]. GLS and GLS response to dipyridamole stress were impaired among patients with angina, non-obstructive CAD, and CMD, reflecting subclinical LV systolic dysfunction and lack of contractile reserve [14]. An association between low GLS and the presence of CMD was seen in women with symptoms of coronary X syndrome [28]—a result not directly comparable to our cohort with only 23% of female patients. Indeed, it has been suggested that the risk factor profile, symptomatology, and prognosis of both HFpEF and microvascular angina differ substantially between genders.

There may be at least two plausible explanations why we did not observe a difference in GLS between CMD patients and those with normal microcirculation. Firstly, not all methods of coronary circulation assessment are equally reliable. The direct, invasive measurements are judged more accurate than indirect, non-invasive methods. In particular, the feasibility and quality of LAD flow measurement in TTE (used in many cited studies) depend substantially on technical conditions and operator experience. Measuring only CFR—this method does not discern between flow limited by epicardial artery stenosis and truly impaired microcirculation reactiveness—is easily assessed by invasive measurement of IMR performed in our study. Secondly, the population of our patients is characterized by a relatively high prevalence of cardiovascular risk factors and known (and previously treated) CAD. Therefore, the group with normal microcirculatory indices cannot be thought of as completely free of cardiovascular disease. In many patients, there might have been changes in myocardial structure and function that affect the measured parameters. Consequently, a potential difference in strain values between patients with no cardiovascular disorders and those with impaired coronary microcirculation might not be visible in our study due to other confounding factors. It is well conceivable that patients assessed by noninvasive methods in other studies are characterized by fewer cardiovascular comorbidities than those referred to the cathlab.

Interestingly, studies of STEMI patients might suggest that there is no direct relationship between GLS and IMR. Normalization of both IMR and LV function (including GLS) has been reported [29], while some therapies improved only LV contractility with IMR remaining high [30].

### 4.3. Left Atrial Strain

In our study group, we observed no difference in PALS or PACS between patients with normal and elevated IMR. Nevertheless, the average PACS and especially PALS in the normal IMR group were somewhat lower than the values usually reported as mean for the healthy [31]. It can be argued therefore that even if there is indeed an association between left atrial strain and CMD, it might have been masked in our study by other cardiovascular risk factors affecting left atrium in the patients with normal IMR.

An elevated microvascular resistance was associated with lower LA strain values in patients without obstructive CAD by Keulards et al. [23]. Diastolic dysfunction was also associated with high microvascular resistance, but in some cases the high IMR and low LA strain pattern was seen in patients with normal diastolic function. This was interpreted by the investigators as revealing changes in microvasculature and left atrial function that preceded full-scale diastolic dysfunction. This study offers a more direct comparison to our research, as IMR was measured invasively (although using a different catheter and a different vasodilation protocol). It may be hypothesized that our study population represents a different pattern of diastolic dysfunction (elevated E/E′ values) coexisting with high microvascular resistance but not with impaired left atrial strain.

### 4.4. CAD Risk Factor Profile

In our study, there was no statistically significant correlation between classic clinical and biochemical CAD risk factors and high microvascular resistance. This observation is similar to the findings of Kobayashi et al., where only diabetes and—somewhat surprisingly—low cholesterol levels were associated with high IMR [32]. The established cardiovascular risk factors might not be good predictors of high IMR, and, consequently, until other specific revelators of CMD are found, patients with clinical suspicion of microcirculation impairment require invasive physiological assessment of coronary circulation. On the other hand, our findings of significantly higher LDL levels associated with low CFR and a trend towards higher LDL in patients with high IMR might suggest a role of hypercholesterolemia in the development of microvascular dysfunction. There is emerging evidence that dyslipidemia might be one of the factors predisposing to the development of CMD [33], and there have been studies where treatment with statins reduced periprocedural microvascular dysfunction during PCI [34]. As for a more pronounced correlation of abnormal CFR (than IMR) with dyslipidemia, it could be tentatively suggested that—as CFR depends both on the flow in large vessels and microcirculation—subclinical stages of coronary atherosclerosis in those patients affect large vessel reactivity, which adds to the effect of CMD.

When comparing our results with other studies on echocardiographic parameters of CMD patients, two important facts should be taken into consideration. Firstly, various methods have been used to diagnose CMD (only a small fraction of studies rely on invasive measurements). Secondly, the selection of patients to be investigated varied significantly across published studies. It may be argued that our observations offer an example of “true” cathlab patients’ population, that is, with a high incidence or probability of macrovascular CAD and established cardiovascular risk factors. This subset might well be affected by increased microvascular resistance in quite a different way from patients with “pure” (i.e., without overt CAD) HFpEF or INOCA that constituted a large proportion of subjects in other studies. Indeed, many experts underline that both CMD and HFpEF are “umbrella terms” that actually include many subgroups of diverse etiologies and clinical presentation patterns. Such reasoning might explain why not all observations reported by other authors are confirmed by data from our study.

Most authors agree on the plausible explanation that both CMD and abnormal left ventricular and left atrial strain may reflect the earliest stages in the development of HFpEF. Our observation of normal LV and atrial strain coexisting with markers of early LV diastolic dysfunction in patients with high IMR might offer another insight into the hypothesis described above. It may also point to the existence of different subsets of such patients and possibly different stages of the pathology, where various (specific for each subset/stage) echocardiographic parameters are associated with abnormal microvascular function. Therefore, the presented study should primarily be regarded as exploratory and hypothesis-generating.

### 4.5. Limitations

Our sample represents a single center experience and a relatively small number of patients (but comparable to many other single center studies of CMD). The patient group was fairly heterogenous, with a considerable prevalence of cardiovascular risk factors and, in many cases, previously diagnosed (and treated) CAD. Hypothetical factors associated with “pure” elevated IMR might not be clearly detectable in a cohort where comorbidities may blur the view. On the other hand, such a population represents “real life” patients with CMD incidence reflecting clinical practice, possibly different from studies with more restrictive inclusion criteria. We assessed only one type of microvascular impairment (endothelium-independent). On the other hand, the thermodilution method is the most direct evaluation of coronary flow (and its changes), whereas with the non-invasive indirect methods, the measurements are more prone to be influenced by equipment—and investigator—dependent factors.

## 5. Conclusions

A relationship between impaired coronary microvascular function and early echocardiographic changes associated with HFpEF has recently been investigated in many studies. Our finding of elevated E/E′ values and normal LV and LA strain in patients with invasively determined coronary microvascular dysfunction might suggest possible co-development of CMD and early stages of left ventricular diastolic dysfunction. It also implies that in clinical practice, testing for impaired microvessel function may be considered in patients with angina and elevated LV filling pressure. The prognosis and optimal therapy in cases where those conditions coexist remain to be determined by further studies.

## Figures and Tables

**Table 1:** Distribution of quantitative variables—echocardiography measurements, blood test results, and invasive assessment of coronary physiology.

Parameter	Mean	SD
GLS	17.43	3.81
PALS	28.04	6.13
PACS	13.91	3.53
LVd [mm]	51.25	4.77
IVS [mm]	12.15	1.67
LA [mm]	39.71	3.76
LAV [mL]	75.43	11.95
LAVI [mL/m^2^]	36.07	2.43
LA stiffness index	0.28	0.10
EF s [%]	56.75	6.87
LDL [mg%]	85.26	42.28
BMI [kg/m^2^]	28.64	4.78
eGFR [mL/min]	82.66	23.3
HbA1c [%]	6.51	1.23
NT proBNP [pg/mL]	549.7	920.75
IMR	28.3	16.81
CFR	2.76	1.64

**Table 2:** Echocardiographic parameters and cardiovascular risk factors in groups with normal and elevated IMR.

	IMR		W Test
Parameter	<25	≥25	
	A	B	*p*
GLS	17.01 (±3.29)	17.92 (±4.35)	0.21
PALS	27.60 (+6.62)	28.68 (±5.41)	0.7
PACS	13.49 (±3.59)	14.55 (±3.44)	0.35
LVd [mm]	51.74 (±5.10)	50.62 (±4.32)	0.36
IVS [mm]	11.92 (±1.24)	12.44 (±2.09)	0.5
E/E′	6.91 (±2.22)	7.43 (±2.23)	0.36
LAVI [ml/m^2^]	36.46 (±2.090	35.76 (±2.63)	0.46
LA stiffness index	0.27 (±0.11)	0.28 (±0.10)	0.53
EF [%]	57.55 (±5.04)	58.54 (±3.75)	0.46
LDL [mg%]	75.37 (±37.02)	96.64 (±44.38)	0.06
BMI [kg/m^2^]	27.77 (±4.10)	29.39 (±5.48)	0.29
eGFR [mL/min]	85.24 (±19.83)	79.47 (±27.23)	0.52
CRP	3.22 (±6.80)	2.80 (±3.33)	0.46
HbA1c [%]	6.50 (±1.13)	6.52 (±1.40)	0.89
NT pro BNP [pg/mL]	393.53 (±672.15)	430.41 (±715.38)	0.77

**Table 3:** Echocardiographic parameters and cardiovascular risk factors in groups with normal and reduced CFR.

	CFR		W Test
Parameter	≥2	<2	
	A	B	*p*
GLS	17.12 (±3.51)	18.04 (±4.37)	0.34
PALS	27.48 (±6.26)	29.08 (±5.91)	0.37
PACS	13.96 (±3.22)	13.80 (±4.19)	0.76
LVd [mm]	51.54 (±5.07)	50.67 (±4.13)	0.69
IVS [mm]	12.24 (±1.88)	11.94 (±1.16)	0.9
E/E′	6.69 (±1.99)	8.64 (±2.36)	0.016
LAVI [mL/m^2^]	36.03 (±2.64)	36.22 (±2.03)	0.83
LA stiffness index	0.32 (±0.12)	0.26 (±0.10)	0.21
EF [%]	58.14 (±4.41)	57.67 (±4.83)	0.82
LDL [mg%]	72.70 (±27.15)	110.56 (±54.17)	0.019
BMI [kg/m^2^]	28.9 (±4.8)	27.72 (±4.84)	0.26
eGFR [mL/min]	83.14 (±21.70)	81.56 (±27.22)	0.79
CRP	2.36 (±2.88)	4.56 (±8.75)	0.45
HbA1c [%]	6.38 (±1.06)	6.80 (±1.60)	0.26
NT proBNP [pg/mL]	251.71 (±262.12)	727.65 (±1078.98)	0.21

**Table 4:** ROC chart data.

	Variable: E/E′					
	AUC	SE	AUC Lower 95%	AUC Upper 95%	Z = (v1 − 0.5)/v2	*p*
1	0.769	0.105	0.563	0.974	2.564	0.0103

## Data Availability

The raw data supporting the conclusions of this article will be made available by the authors on request.

## Abbreviations

HFpEF	heart failure with preserved ejection fraction
INOCA	ischemia with non-obstructed coronary arteries
CAD	coronary artery disease
LAD	left anterior descending artery
CMD	coronary microvascular dysfunction
IMR	index of microvascular resistance
CFR	coronary flow reserve
LV	left ventricle
LA	left atrium
LAVI	left atrial volume index
PACS	peak atrial contraction strain
PALS	peak atrial longitudinal strain
GLS	global longitudinal strain
